# Reply: Is nesting addiction medicine and hepatology care in the outpatient setting worthwhile? A retrospective case series at a single tertiary center

**DOI:** 10.1097/HC9.0000000000000556

**Published:** 2024-11-25

**Authors:** Rachael Mahle, Paige McLean Diaz, Chantelle Marshall, Russell P. Goodman, Esperance Schaefer, Jay Luther

**Affiliations:** 1 MGH Alcohol Liver Center, Massachusetts General Hospital, Harvard Medical School, Boston, Massachusetts, USA; 2 Division of Gastroenterology, Massachusetts General Hospital, Harvard Medical School, Boston, Massachusetts, USA

We congratulate Gupta and colleagues for their study evaluating the impact of an outpatient integrated model incorporating addiction medicine and hepatology care for patients with alcohol use disorder (AUD). Their results add to the growing evidence that involvement of hepatology care for patients with AUD may improve alcohol therapy engagement.^1^ These findings are particularly important given the fact that most patients with AUD do not receive therapy targeting their alcohol use,^2^ which is the best-studied strategy to prevent the development and progression of alcohol-associated liver disease.^3^ As these integrated models become more established, it will also be important to evaluate the long-term impact of integrated models on liver-related outcomes as well, including progression to cirrhosis, frequency of hepatic decompensation events, transplant eligibility, posttransplant relapse rates and outcomes, and liver-related mortality. Implementing and expanding access to these care models will require a systems-level approach, but the growing body of literature to support an integrated, multidisciplinary approach to AUD and alcohol-associated liver disease care emphasizes the utility.
